# Rapid capillary gel electrophoresis analysis of human milk oligosaccharides for food additive manufacturing in-process control

**DOI:** 10.1007/s00216-020-03119-0

**Published:** 2021-02-08

**Authors:** Marton Szigeti, Agnes Meszaros-Matwiejuk, Dora Molnar-Gabor, Andras Guttman

**Affiliations:** 1 Horváth Csaba Memorial Laboratory of Bioseparation Sciences, Research Center for Molecular Medicine, Faculty of Medicine, Doctoral School of Molecular Medicine, University of Debrecen, Debrecen, 4032 Hungary; 2 Translational Glycomics Group, Research Institute of Biomolecular and Chemical Engineering, University of Pannonia, Veszprem, 8200 Hungary; 3 Glycom A/S, 2970 Hørsholm, Denmark

**Keywords:** Human milk oligosaccharides, In-process control, Capillary gel electrophoresis

## Abstract

Industrial production of human milk oligosaccharides (HMOs) represents a recently growing interest since they serve as key ingredients in baby formulas and are also utilized as dietary supplements for all age groups. Despite their short oligosaccharide chain lengths, HMO analysis is challenging due to extensive positional and linkage variations. Capillary gel electrophoresis primarily separates analyte molecules based on their hydrodynamic volume to charge ratios, thus, offers excellent resolution for most of such otherwise difficult-to-separate isomers. In this work, two commercially available gel compositions were evaluated on the analysis of a mixture of ten synthetic HMOs. The relevant respective separation matrices were then applied to selected analytical in-process control examples. The conventionally used carbohydrate separation matrix was applied for the in-process analysis of bacteria-mediated production of 3-fucosyllactose, lacto-*N*-tetraose, and lacto-*N*-neotetraose. The other example showed the suitability of the method for the in vivo in-process control of a shake flask and fermentation approach of 2′-fucosyllactose production. In this latter instance, borate complexation was utilized to efficiently separate the 2′- and 3-fucosylated lactose positional isomers. In all instances, the analysis of the HMOs of interest required only a couple of minutes with high resolution and excellent migration time and peak area reproducibility (average RSD 0.26% and 3.56%, respectively), features representing high importance in food additive manufacturing in-process control.

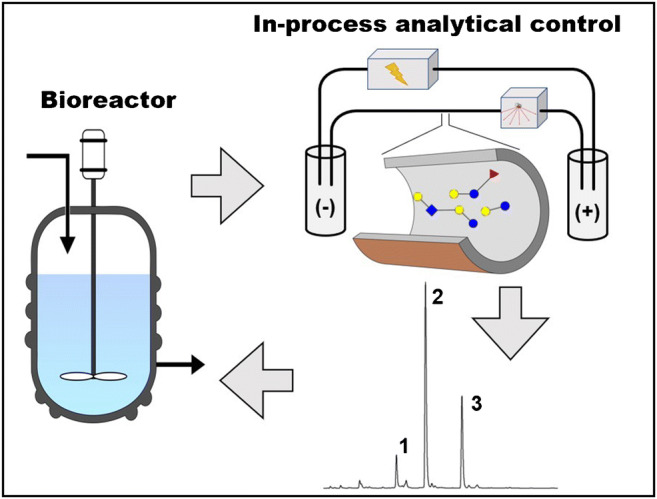

## Introduction

Human milk oligosaccharides (HMOs) are the third main ingredients in breastmilk after lactose and lipids [1]. HMOs play a significant role in health benefits such as infant gut microbiota [2], cognitive brain function, and immunity development [3–6]. Infants receiving exclusively breastmilk for the first 6 months of their lives apparently show less likelihood of various infections, respiratory illnesses, and diarrhea [7]. Undoubtedly, HMOs are offering various health benefits to infants, but it may also be beneficial for older children as well as for adults [8, 9].

HMOs are unconjugated glycans typically found in concentrations of 5–20 g/L in mother’s milk [10]. This high concentration and structural diversity of these short but complex carbohydrates are unique to humans. For the time being, more than 200 HMOs have been identified of which more than 140 were structurally elucidated [1, 11, 12]. HMOs are usually composed of 3 to 10 monosaccharide units and can all be catogorized in three groups: core, fucosylated, and sialylated. The building blocks of these oligosaccharides are glucose (Glc), galactose (Gal), *N*-acetyl-glucosamine (GlcNAc), fucose (Fuc), and sialic acid (*N*-acetylneuraminic acid, Neu5Ac). Lactose is considered as the simplest HMO core structure that can be further decorated with fucose and/or sialic acid residues. Linear (β1-3) or branched (β1-6) oligosaccharides are the result of elongation of lactose with lacto-*N*-biose and/or *N*-acetyllactosamine [11, 13]. The HMO composition differs from mother to mother and the concentrations change over the lactation course [14–17]. Within the group of neutral HMOs lacto-*N*-tetraose (LNT), lacto-*N*-*neo*-tetraose (LNnT) are considered as core structures, and the fucosylated forms of 2′-fucosyllactose (2**′**-FL), 3-fucosyllactose (3-FL), 2′,3-difucosyllactose (DFL or LDFT), lacto-*N*-fucopentaose I (LNFP-I), lacto-*N*-fucopentaose II (LNFP-II), lacto-*N*-fucopentaose III (LNFP-III), lacto-*N*-fucopentaose V (LNFP-V), lacto-*N*-difucohexaose I (LNDFH-I), and lacto-*N*-difucohexaose II (LNDFH-II) are frequently occuring compositions. The most abundant sialylated HMOs are 3′-sialyllactose (3**′**-SL), 6′-sialyllactose (6**′**-SL), sialyl-lacto-*N*-tetraose a (LST-a), sialyl-lacto-*N*-tetraose b (LST-b), and sialyl-lacto-*N*-*neo*-tetraose c (LST-c), just to mention the most important ones [17–21].

To make HMOs broadly accessible as, e.g., infant formula additives, their production has become a focus for a number of companies and research groups. HMOs can be produced chemically and by biotechnology approaches in vitro or in vivo. For large scale production, their chemical synthesis is the least economic and environmentally friendly approach, since it includes multi-step protection/deprotection reactions with the use of large amounts of organic solvents. The in vivo biotechnology approach is based on genetically engineered (via knock-outs and essential gene inserts) bacterial host organisms to produce human milk oligosaccharides by utilizing lactose to express the desired product. It has been shown that this approach is readily scalable even up to multi-ton production. In vitro technologies are using single HMOs to produce more complex ones by means of adding the appropriate enzymes into the reaction, more specifically transglycosylases to the donor-acceptor substrates, while lactose is released together with the product HMO after reaching equilibrium. These enzymes are specifically engineered for these purposes since they are rarely found in nature. In vitro production requires the supply of the corresponding HMO donor(s) and acceptor(s) [21–28].

Regardless of the manufacturing process chosen, in-process control (IPC) plays an important role during production. Close monitoring of the selected products allows timely modification of the process parameters to reach optimal production. Therefore, a desirable IPC method should be fast, reliable, robust, and easy to conduct, providing the required information in the timeframe of the process. Since the scientific and industrial interest towards these compounds have been significantly increased in recent years, it called for a rapid development of suitable analytical methods. A great number of existing techniques for qualifying and quantifying HMOs have been recently summarized in [29]. Separations can be carried out by either liquid chromatography using hydrophilic interaction (HILIC), graphitized carbon or anion-exchange (HPAEC)–based columns, or by electric field–mediated separation techniques such as capillary electrophoresis (CE) or microchip electrophoresis (ME). Liquid chromatographic methods can be coupled with detection systems like evaporative light-scattering (ELSD), mass spectrometer (MS), refractive index (RI), fluorescent (FL), ultra-violet (UV), pulsed-amperometric (PAD), or charged aerosol detection (CAD). MS, CAD, ELSD, and RI can detect HMOs without labeling. These latter two detectors are limited by the use of gradients with lower sensitivity than that of MS and CAD. For fluorescent (FL) or UV detection, a derivatization or labeling step is necessary since most HMOs are non-UV or fluorescently active.

Capillary electrophoresis–based separation methods are not widespread yet in the field of HMO analysis. Due to the lack of charge on most oligosaccharides (e.g., non-sialylated), electric field–mediated analysis techniques had to utilize various labeling methods. Accordingly, using either non charged tagging for acidic sugars [30], or charged fluorescent labeling agents such as 3-aminobenzoic acid for neutral oligosaccharide analysis [31] and 2-aminoacridone or 8-aminopyrene-1,3,6-trisulphonic acid (APTS) for total HMO analysis [32, 33] is recommended. It is important to note that in CE, the resolution between the analyte components can be increased by using longer capillaries if separation time is not an issue. Alternatively, different separation buffers and gels can be employed to obtain enhanced resolution in a reasonable time frame [29, 34]. Furthermore, conventional oligosaccharide sample preparation techniques—such as labeling—required overnight steps that were undesirable during process control applications in biotechnology settings. One of the most commonly used fluorescent labels, APTS, provided both excellent fluorescent characteristics and the necessary charge for the electromigration with as short as 1-h derivatization time, i.e., readily suitable for routine process analytical applications [35–40].

In this study, novel CE-based methods were developed for in-process control analytical applications to follow-up bioprocessing-mediated HMO production. The introduced methods are short and highly reproducible, enabling rapid measurement of the main components and its related impurities. Two gel-buffer types were chosen to fulfill the required separation criteria. Based on their resolution characteristics for specific HMOs, they were employed in different in-process control examples. The samples analyzed in this study were from specific fermentation processes in HMO production using modified *E. coli* in the bioreactor.

## Materials and methods

### Chemicals and reagents

Acetic acid (glacial), tetrahydrofuran (THF), sodium-cyanoborohydride (1 M in THF), HPLC grade water, and all other chemicals were from Sigma–Aldrich (St. Louis, MO, USA). The separation gel-buffer systems of the Fast Glycan Sample Preparation and Analysis kit (HR-NCHO) and the SDS-MW kit (CE-SDS) as well as the 8-aminopyrene-1,3,6-trisulfonic acid (APTS) were from Sciex (Brea, CA). The HMO standards (Table 1) and the biotechnology process analytical samples were all provided by Glycom A/S (Hørsholm, Denmark).

**Table 1:**
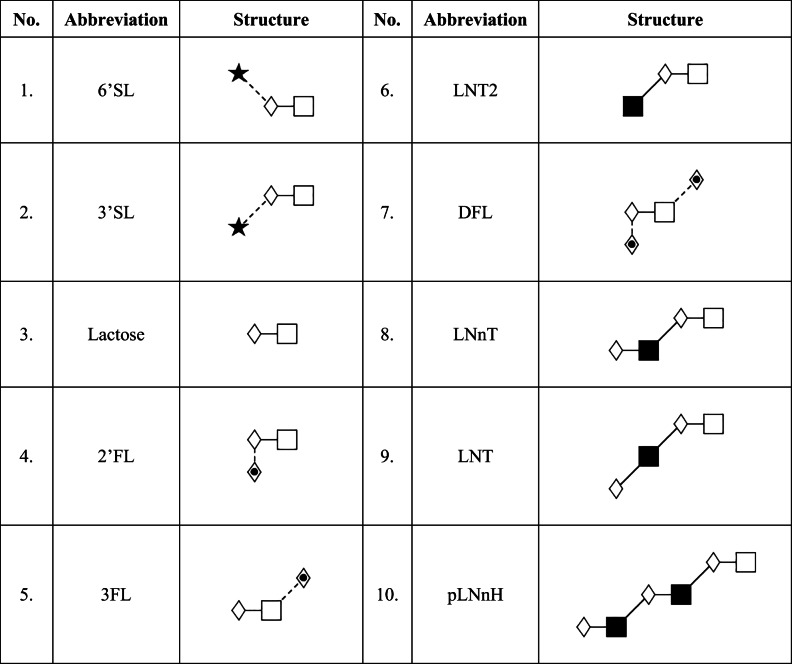
Synthesized human milk oligosaccharides used in the study. Abbreviated glycan structural names followed the nomenclature proposed by Harvey et al. [41]*.* **Symbols:** *square* – D-glucose; *filled square* – GlcNAc; *diamond* – D-galactose; *diamond with dot* – fucose; *filled star* – sialic acid

### Sample preparation

The standard and IPC samples were prepared as follows: 30 μL of HPLC grade water and 20 μL of labeling solution (2.4 mM of APTS and 40 mM NaBH_3_CN in 20% acetic acid) were added to 2.0 mg of each sugar standards. The reaction mixture was incubated with the open vial lid approach as described in [42], followed by reconstitution in 100-μL water. The standard stock solutions were mixed in equal amounts and diluted to 1000-fold in HPLC grade water prior to CGE-LIF analysis. For limit of detection (LOD) and limit of quantitation (LOQ) analysis, the stock solutions were double diluted from 1× to 1024× fold.

### Capillary gel electrophoresis

A PA800 Plus Pharmaceutical Analysis system (Sciex) equipped with a solid-state laser-induced fluorescence detector (488-nm excitation wavelength with 520-nm emission filter) was employed for all capillary electrophoresis separations with the separation gel-buffer systems of HR-NCHO (high resolution N-linked carbohydrate) buffer commonly used for glycan separation and SDS-MW (sodium dodecyl sulfate–molecular weight) buffer designed for protein analysis (both from Sciex). CGE-LIF analysis of the APTS labeled HMOs and biotechnology process analytical samples were performed in 20-cm effective length (30-cm total length), 50-μm ID bare fused silica capillary columns by applying 1000 V/cm electric field strengths in reversed polarity mode (cathode at the injection side). The applied buffers were pre-filled into the capillary prior each analysis with no conditioning steps between runs in case of the HR-NCHO buffer and using 0.1-M NaOH and HPLC grade water wash (both at 80 psi for 2.0 min) in case of the use of the SDS-MW buffer. Electrokinetic sample injection protocol: first 5.0 psi for 5.0-s water pre-injection, followed by 2.0 kV for 2.0-s sample injection. Data acquisition and analysis were accomplished using the 32Karat (version 10.1) software package (Sciex).

## Results

Two different gel-buffer systems were evaluated for high resolution capillary electrophoresis analysis of a mixture of 10 synthetic human milk oligosaccharides. The appropriate methods were then applied for in-process analytical control for 2′- and 3-fucosyllactose (2′FL and 3FL) as well as lacto-*N*-tetraose (LNT) and lacto-*N*-neotetraose (LNnT) production. The HR-NCHO separation matrix [43] has been developed for the analysis of APTS-labeled N-linked oligosaccharides of biological origin, while the SDS-MW separation gel was developed for CGE-based protein sizing. The high boric acid content of the latter [44] proved to be particularly useful for HMO analysis by exploiting the separation enhancing effect of borate-vicinal OH interactions [45].

### Evaluation of the separation matrices

**Fig. 1:**
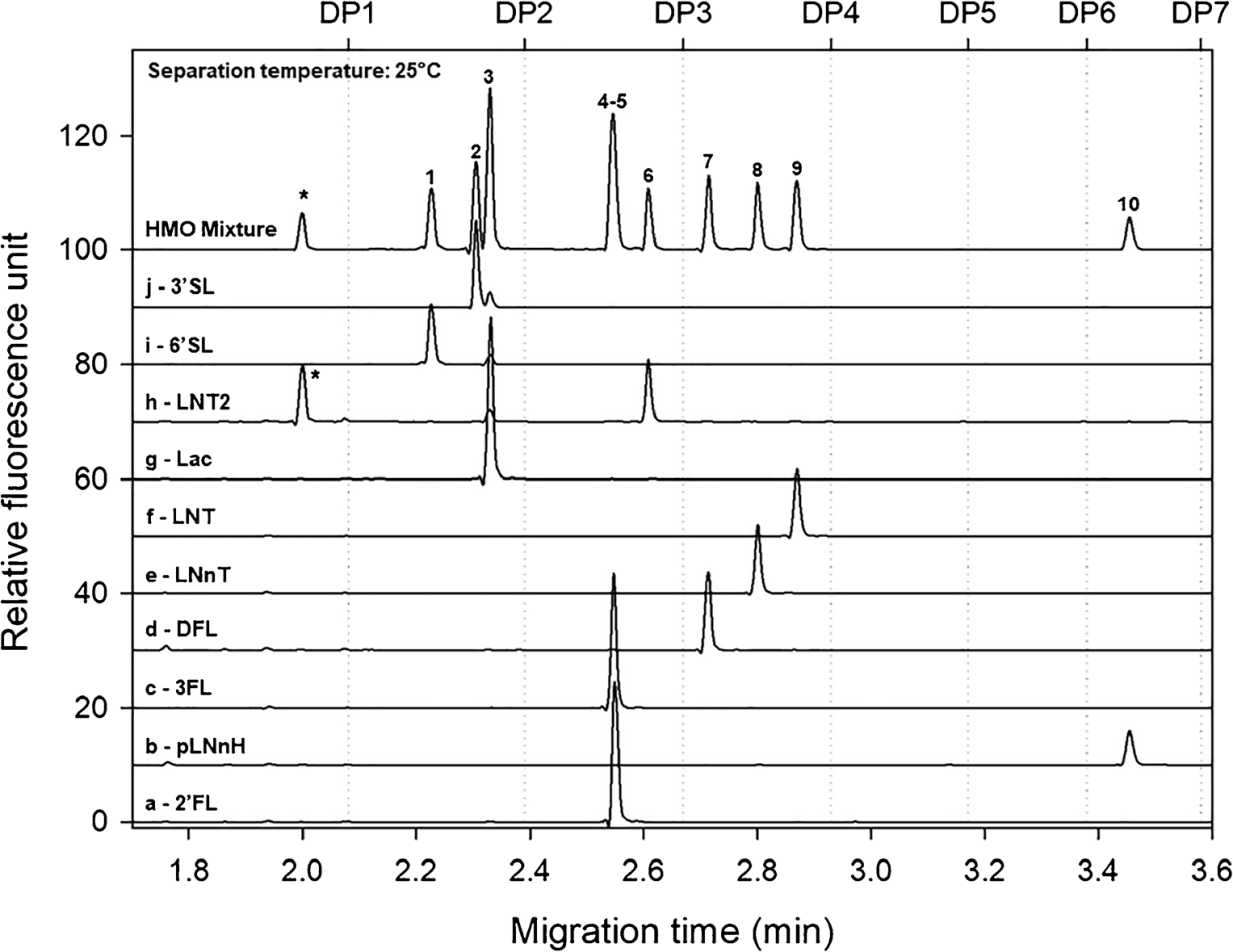
Capillary gel electrophoresis analyses of the APTS-labeled synthesized HMOs (traces a-j) and their mixture (trace HMO mix) using the conventional carbohydrate separation gel-buffer system. Peaks: 1, 6′SL; 2, 3′SL; 3, Lac; 4, 2′FL; 5, 3FL; 6, LNT2; 7, DFL; 8, LNnT; 9, LNT, 10, pLNnH; and the asterisk (*), *N*-acetylglucosamine. Conditions: bare fused silica capillary with 20-cm effective length (30-cm total length), 50-μm ID; HR-NCHO gel matrix; separation temperature: 25 °C; applied separation potential: 30 kV; injection sequence: (1) 5.0 psi for 5.0-s water pre-injection, (2) 2.0 kV for 2.0-s sample injection

Figure 1 compares the CGE-LIF analysis results of the individual HMO standards and their mixture using the HR-NCHO separation gel-buffer system. According to their charge to hydrodynamic volume ratios, the extra charge carrying sialylated structures migrated first (peaks 1 and 2), followed by the neutral tri, tetra, and hexasaccharides. As one can observe, this gel-buffer system well separated all synthesized HMOs except the fucosyllactose positional isomers of 2′FL and 3FL. The peak with the asterisk (*) depicts the monosaccharide *N*-acetylglucosamine decomposition product/impurity of the lacto-*N*-triose (traces h and HMO Mixture). Please note that the entire analysis time including the slowest migrating pLNnH peak was less than 3.5 min at 25 °C separation temperature.

**Fig. 2:**
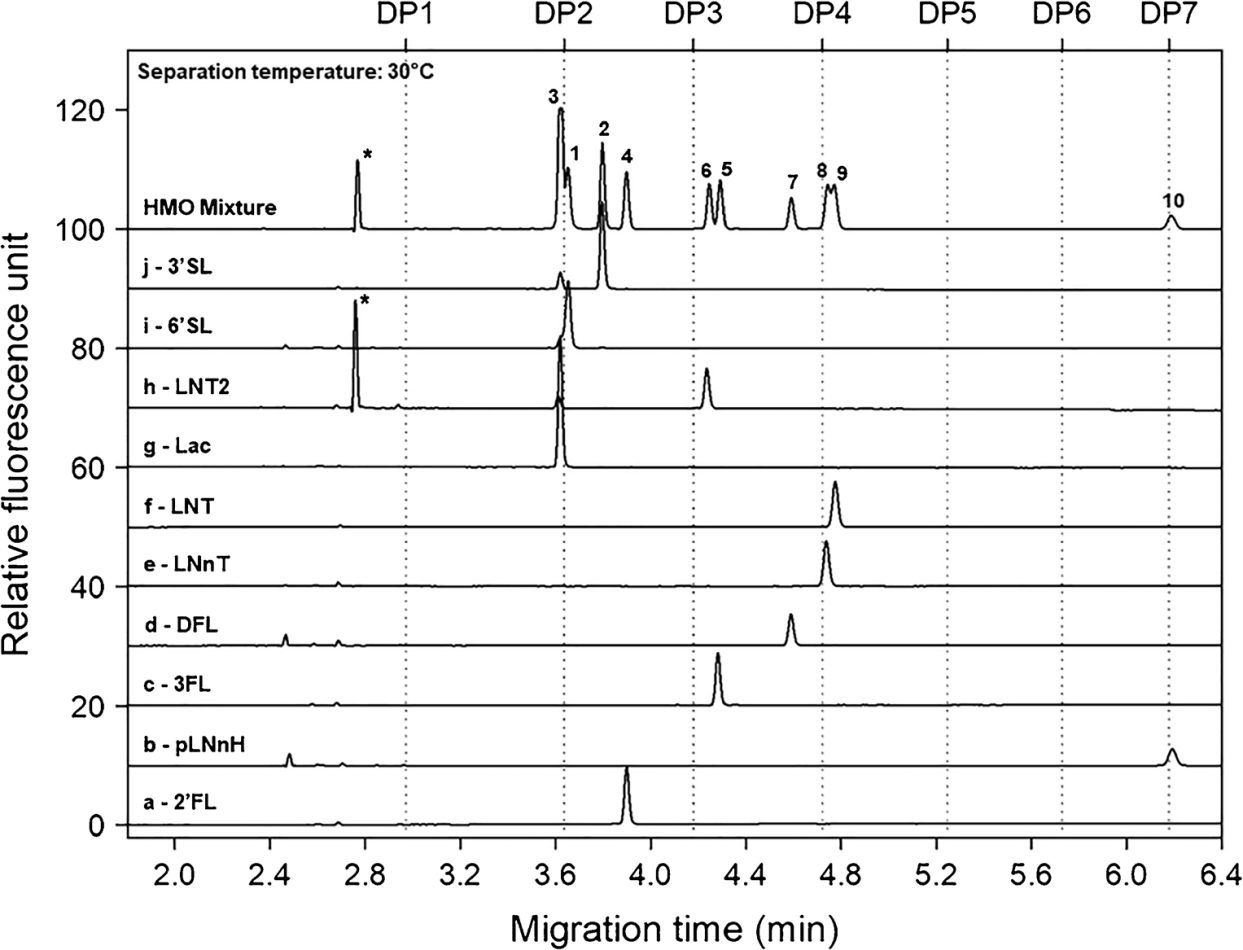
Capillary gel electrophoresis separations of the APTS-labeled synthesized HMOs (traces a-j) and their mixture (trace HMO mix) using a high borate containing gel-buffer system. Peaks: 1, 6′SL; 2, 3′SL; 3, Lac; 4, 2′FL; 5, 3FL; 6, LNT2; 7, DFL; 8, LNnT; 9, LNT; 10, pLNnH; and the asterisk (*), *N*-acetylglucosamine. Conditions: same as in Fig. 1 except the separation matrix was the SDS-MW gel-buffer system and the separation temperature was 30 °C

Next, the high boric acid–containing SDS-MW gel-buffer system was evaluated. As one can observe in Fig. 2, with the use of this gel-buffer system, the individual HMOs and the components of the mixture migrated quite differently. This phenomena was assumable due to their complexation with the high boric acid content (~ 0.6 M) of the background electrolyte, enhancing the separation via transitional diol-borate adduct formation. Due to this complexation phenomena, the sialylated trisaccharides of 6′SL and 3′SL (peaks 1 and 2) migrated somewhat slower that of the lactose disaccharide (peak 3). However, the previously co-migrating (Fig. 1) 2′FL (peak 4) and 3FL (peak 5) were separated so well that the LNT2 structure was even migrating in between (peak 6). Interestingly, on the other hand, the tetrasaccharide positional isomers of LNnT (peak 8) and LNT (peak 9) were only marginally separated (split peak), probably also caused by the borate complexation phenomena. Similar to as of above, the hexasaccharide pLNnH migrated last in this system as well at 6.2 min at 30 °C separation temperature.

### Statistical analysis

A comprehensive statistical evaluation was performed using the two separation media introduced under “Evaluation of the separation matrices” (Figs. 1 and 2). Intraday and interday migration time, peak area, peak area %, and resolution reproducibilities were compared using six consecutive injections. The values obtained were also compared during calibration, limit of detection (LOD), and limit of quantitation (LOQ) determination for each carbohydrate structures in the test mixture. Please note that with electrokinetic injection, while the response to the injection parameters was linear, the response to the sample concentration exhibited exponential change as demonstrated earlier [46]. However, due to the high viscosity of both separation media, electrokinetic injection was necessary since pressure injection into the high viscosity gels resulted in weak signal and some peak broadening. Good correlations were found between the actual sugar sample concentrations and the resulting peak areas, as shown in Tables 2 and 3.

**Table 2:** Statistical evaluation of HMO analysis by capillary electrophoresis – HR-NCHO gel buffer

Separation matrix: HR-NCHO gel			Average %RSD (9 peaks)					
			Migration time			Area	%Area	Resolution
Intraday reproducibility			0.310			2.383	0.695	1.799
Interday reproducibility			0.317			2.860	1.460	2.505
Dilution calibration (1×–1024×)			0.208			3.164	2.001	3.496
Peak	HMO	Calibration				LOD (μg/mL)	LOQ (μg/mL)	Resolution
		*r*^2^	Equation	*a* std. error	*b* std. error			
1	6′SL	0.9997	*y* = 118.5548 · (1 − *e*^−0.0327*x*^)	2.9269	0.0015	3.79E-03	1.26E-02	–
2	3′SL	0.9997	*y* = 159.5336 · (1 − *e*^−0.0337 · *x*^)	4.0393	0.0016	2.77E-03	9.23E-03	3.19
3	Lac	0.9996	*y* = 316.8944 · (1 − *e*^−0.0323 · *x*^)	9.4069	0.0018	1.49E-03	4.96E-03	1.21
4	2′FL & 3FL	0.9995	*y* = 294.4801 · (1 − *e*^−0.0313 · *x*^)	9.7998	0.0019	1.61E-03	5.36E-03	7.85
5	LNT2	0.9996	*y* = 102.2763 · (1 − *e*^−0.0315 · *x*^)	3.0573	0.0017	4.67E-03	1.55E-02	2.04
6	DFL	0.9994	*y* = 137.7410 · (1 − *e*^−0.0321 · *x*^)	4.6478	0.0020	3.57E-03	1.19E-02	3.95
7	LNnT	0.9994	*y* = 124.2720 · (1 − *e*^−0.0320 · *x*^)	4.2193	0.0020	3.98E-03	1.33E-02	3.06
8	LNT	0.9994	*y* = 141.8412 · (1 − *e*^−0.0322 · *x*^)	5.0280	0.0021	3.53E-03	1.18E-02	2.39
9	pLNnH	0.9989	*y* = 72.7210 · (1 − *e*^−0.0327 · *x*^)	3.3270	0.0028	7.44E-03	2.48E-02	19.44

**Table 3:** Statistical evaluation of HMO analysis by capillary electrophoresis – SDS-MW gel buffer

Separation matrix: SDS-MW gel				Average %RSD (9 peaks)				
				Migration time		Area	%Area	Resolution
Intraday reproducibility				0.316		4.268	1.803	2.963
Interday reproducibility				0.383		4.735	3.243	3.963
Dilution calibration (1×–1024×)				0.285		4.922	3.974	4.418
Peak	HMO	Calibration				LOD (μg/mL)	LOQ (μg/mL)	Resolution
		*r*^2^	Equation	*a* std. error	*b* std. error			
1	Lac	0.9993	*y* = 838.8356 · (1 − *e*^−0.0202 · *x*^)	16.0734	0.0027	4.24E-04	1.41E-03	–
2	6′SL	0.9995	*y* = 181.9167 · (1 − *e*^−0.0300 · *x*^)	3.5602	0.0011	2.91E-03	9.70E-03	0.55
3	3′SL	0.9999	*y* = 321.6354 · (1 − *e*^−0.0243 · *x*^)	9.8706	0.0014	1.28E-03	4.28E-03	3.88
4	2′FL	0.9998	*y* = 278.7293 · (1 − *e*^−0.0201 · *x*^)	7.8926	0.0012	1.80E-03	6.01E-03	2.61
5	LNT2	0.9999	*y* = 218.6504 · (1 − *e*^−0.0197 · *x*^)	5.1687	0.0011	2.37E-03	7.91E-03	8.24
6	3FL	0.9998	*y* = 262.4525 · (1 − *e*^−0.0201 · *x*^)	6.8266	0.0012	1.94E-03	6.46E-03	1.39
7	DFL	0.9996	*y* = 217.9449 · (1 − *e*^−0.0177 · *x*^)	4.3771	0.0011	2.66E-03	8.86E-03	6.59
8	LNnT	0.9994	*y* = 319.3016 · (1 − *e*^−0.0173 · *x*^)	9.7608	0.0011	1.87E-03	6.23E-03	2.82
9	LNT	0.9993	*y* = 336.4311 · (1 − *e*^−0.0162 · *x*^)	9.0343	0.0010	1.92E-03	6.39E-03	0.67
10	pLNnH	0.9992	*y* = 154.6536 · (1 − *e*^−0.0160 · *x*^)	3.6447	0.0011	4.06E-03	1.35E-02	22.60

### In-process analytical control

**Fig. 3:**
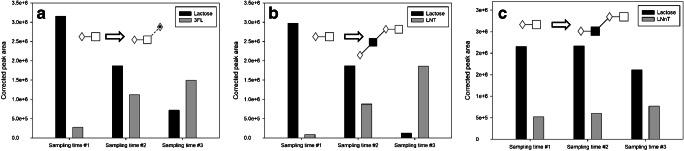
In-process analysis of bacteria-mediated production of 3FL, LNT, and LNnT (panels **a**, **b**, and **c**, respectively) tracked by ultrafast capillary gel electrophoresis. Separation conditions were the same as in Fig. 1

The first in-process analytical control example (Fig. 3) shows the runs of bacteria-mediated production of 3FL, LNT, and LNnT. In this case, the bacteria modified the lactose to the more complex structures of 3FL (panel a) by the addition of a fucose, as well as the LNT and LNnT (panels b and c, respectively) by the addition of an *N*-acetyl-glucosamine and a galactose unit with different linkages. Accordingly, the charts in Fig. 3 demonstrate the timely increase of the final products of 3FL, LNT, and LNnT and concomitant decrease of the lactose starting material during the process. The bars in the diagrams represent the peak areas of the corresponding structures analyzed by capillary gel electrophoresis, in this case using the ultrafast analysis option with the HR-NCHO gel.

**Fig. 4:**
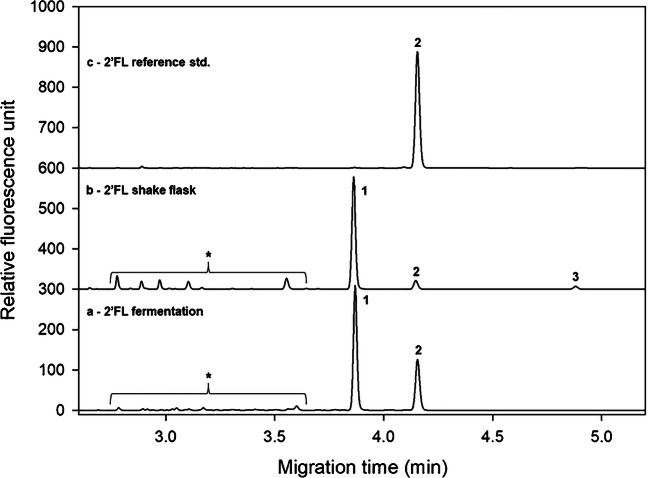
In-process analytical control of the 2′FL fermentation process followed by CGE-LIF. Separation conditions were the same as in Fig. 2. Peaks: 1, lactose; 2, 2′FL; 3, DFL; the asterisk (*), small sugar degradation products and impurities

Another important biotechnology-related application of the rapid CGE-LIF-based sugar analysis protocol is shown in Fig. 4, where the in vivo process using a shake flask (trace b) or a fermentation (trace a) approach for 2′FL production was closely followed by using the SDS-MW gel-buffer system. Peak 1 is the 2′FL reference standard, while peaks 2 and 3 represent the products with single fucosylation (peak 2: 2′FL, Table 1, line 4) and double fucosylation (peak 3: DFL, Table 1, line 7), respectively. The engineered *E. coli* utilized the added lactose and produced 2′FL. In some cases, not only 2′FL was produced, but DFL was also obtained as a by-product due to the type of fucosyltransferase inserted during strain construction. Throughout the fermentation process, the amount of lactose decreased and the 2′FL and DFL concentrations increased as was reported in [27]. In this instance, the SDS-MW gel-buffer system was utilized to detect any possible 3FL formation as another theoretically possible by-product, since that particular matrix offered excellent separation of the 2′FL and 3FL trisaccharides (Fig. 2). Please note that apparently no 3FL was formed in the selected fermentation process.

## Discussion

In this paper, two complementary gel-buffer systems were evaluated for the analysis of a mixture of 10 synthesized human milk oligosaccharides (Table 1). Both separation matrices showed excellent resolution for most of the HMOs in the test mixture. While the utilization of the carbohydrate-specific separation matrix resulted in no separation of the fucosyllactose linkage isomers of 2′FL and 3FL, the high borate concentration SDS-MW gel successfully separated those. The linkage isomers of LNT and LNnT, on the othre hand, were better separated by the carbohydrate-specific gel composition. The methods were then applied to different food additive manufacturing applications. First, it was demonstrated that the conventional carbohydrate separation gel was suitable for the in vivo process analytical control during fermentation with just a few minute separation time. The in vivo production of the analyzed HMOs was based on a whole cell living factory approach, where the production organism was modified to express relevant sugar transferases suitable to transfer the targeted activated sugar nucleotides to the lactose moiety forming the desired HMO product. The capillary gel electrophoresis method applied here consistently identified the decrease of lactose levels and the increase of the products over the processing time. This enabled precise determination of the end point titer, i.e., when the lactose has almost disappeared, hence the production of the target compounds would stop increasing. Another example showed the in vivo process analytical studies of 2′FL fermentation and deep-well assays, respectively. Deep-well assay or shake flask test can be considered as a small-scale or test fermentation, running for a certain period and analyzing the endpoint. Fermentation on the other hand was performed by a standard procedure and was traced over time. The advantages of the CGE-LIF analysis methods applied in the examples shown in this paper were the short separation times (< 6 min) with high resolution between the analytes of interest with excellent intra/interday migration time (RSD = 0.26%) and peak area (RSD = 3.56%) reproducibility, allowing fast and accurate in-process analytical control.

## Abbreviations

GlcNAc: *N*-Acetylglucosamine
LacNAc: *N*-Acetyllactosamine
APTS: 8-Aminopyrene-1,3,6-trisulfonic acid
CGE-LIF: Capillary gel electrophoresis with laser-induced fluorescence detection
DFL: Difucosyllactose
2′FL: 2′-Fucosyllactose
3FL: 3-Fucosyllactose
HMO: Human milk oligosaccharides
IPC: In-process control
LNB: Lacto-*N*-biose
pLNnH: *para*-Lacto-*N*-neohexaose
LNnT: Lacto-*N*-neotetraose
LNT: Lacto-*N*-tetraose
LNT2: Lacto-*N*-triose
Lac: Lactose
3′SL: 3′-Sialyllactose
6′SL: 6′-Sialyllactose
